# Can donor aid for health be effective in a poor country? Assessment of prerequisites for aid effectiveness in Uganda

**Published:** 2009-10-22

**Authors:** Nabyonga Orem Juliet, Ssengooba Freddie, Sam Okuonzi

**Affiliations:** 1 WHO Uganda Country office;; 2 Makerere University School of Public Health,; 3 Makerere University Regional Centre for Quality of Health Care.

**Keywords:** Donor, aid for health, financing, effectiveness, Uganda

## Abstract

**Background:**

Inadequate funding for health is a challenge to attaining health-related Millennium Development Goals. Significant increase in health funding was recommended by the Commission for Macroeconomics and Health. Indeed Official Development Assistance has increased significantly in Uganda. However, the effectiveness of donor aid has come under greater scrutiny. This paper scrutinizes the prerequisites for aid effectiveness. The objective of the study was to assess the prerequisites for effectiveness of donor aid, specifically, its proportion to overall health funding, predictability, comprehensiveness, alignment to country priorities, and channeling mechanisms.

**Methods:**

Secondary data obtained from various official reports and surveys were analyzed against the variables mentioned under objectives. This was augmented by observations and participation in discussions with all stakeholders to discuss sector performance including health financing.

**Results:**

Between 2004–2007, the level of aid increased from US$6 per capita to US$11. Aid was found to be unpredictable with expenditure varying between 174–8722;360 percent from budgets. More than 50% of aid was found to be off budget and unavailable for comprehensive planning. There was disproportionate funding for some items such as drugs. Key health system elements such as human resources and infrastructure have not been given due attention in investment. The government’s health funding from domestic sources grew only modestly which did not guarantee fiscal sustainability.

**Conclusion:**

Although donor aid is significant there is need to invest in the prerequisites that would guarantee its effective use.

## Background

Inadequate funding remains a challenge to scaling up coverage of essential interventions in developing countries and meeting health-related Millennium development goals (MDGs). In the World Health Organization Africa Region (WHO-AFRO) in 2003, per capita government expenditure on health in 35 of the 46 member states was below the recommended US$34 per capita [1–2].

The Commission on Macroeconomics and Health (CMH) recommended significant increases in funding for health as a necessary condition for scaling up coverage with essential interventions. The increase would be on two fronts, low and middle income countries increasing domestic funding and high income countries committing significantly increasing financial assistance in the form of grants [2]. In line with this, donor funding is playing an increasingly significant role in health financing in developing countries [3]. Official developmental assistance for health (DAH) increased from US$10.9 billion in 2001 to US$21.8 billion in 2007 [4]. Significant growth of Global Health Initiatives (GHI) has been witnessed in recent years becoming a major source of funding for health in low-income countries. In 2003 and 2004, the Global Fund against AIDS, Tuberculosis, and Malaria (GFATM) and the Global Alliance for Vaccine Initiative (GAVI) made commitments of approximately US$1 billion [5]. Further commitments were made through debt relief initiatives announced by the World Bank, the Africa Development Bank, and the International Monetary Fund amounting to US$51 billion [6]. Similarly, external resources for health as a percentage of total health expenditure have increased over the years in several developing countries [7].

Donor aid has taken several forms among which are donor projects, Global Health Initiatives (GHIs), programme support and budget support. Several issues have been raised regarding the donor aid, including projects being generally unsuccessful [8], earmarked funding that may not be well aligned to health priorities of recipient countries [9], and poor accountability [10–11]. Donors contributing directly to the national budgets often require that governments increase their social sector spending, a condition often unfulfilled. Many GHIs are centered on specific interventions and funding-specific inputs [12]. Nongovernmental organizations, who are important providers of health care in deprived populations in many African countries, often do not benefit from increased aid flows [13].

Uganda receives donor aid from bilateral and multilateral donors and from GHI. Between 2000 and 2005, the country benefited from the Heavily Indebted Poor Countries (HIPC) initiative and thereafter multi-donor budget support up to now. Uganda also benefited from GAVI since 2001 and from GFATM since 2002–2003. Modalities of channeling donor aid for health include provision of funds to support the government budget which could be general or earmarked for the health sector and provision of aid as project support which could be on budget (reflected in the Medium Term Expenditure Framework) or off budget.

In line with global commitments, there has been a shift in the preferred method of delivering aid in Uganda. Several donors and the government have clearly expressed preference for donor budget support as opposed to supporting specific projects [14]. This is meant to offer better alignment to government priorities as allocations are made within government budget systems. It also means that flexibility in resource use is more likely to be ensured and that expenditures are monitored more closely.

Discussions on improving aid effectiveness have taken place through the High-level Forum on Harmonization in Rome (2003), the Marrakech Round Table on Managing for Development results (2004), the Paris Declaration on Harmonization and Alignment (2005), and the International Health Partnership and related initiatives (iHP+) (2007). A commitment was made to harmonize and align aid, emphasizing the use of country frameworks and processes, increasing alignment of aid with partner countries’ strategies, eliminating duplication of efforts, and minimizing transaction costs [15]. The Government of Uganda (GoU) fully subscribes to global efforts to make aid more effective as specified in the Paris Declaration agenda and signed on to the IHP+ in February 2009.

For donor aid to be effective several prerequisites must be in place, including fiscal sustainability of the wider reform programme, sustainability of aided development projects, predictability, fungibility, and absorptive capacity [16]. Other preconditions that must be in place include local ownership of development strategies and effective stewardship of governments, improved donor coordination, stronger partnerships, results-based approach, capacity building, and engaging civil society. Equally important is the commitment from donors to align to government plans and use government structures [17]. Although civil society organizations are important providers of health care and significant proportions of donor funds are channeled through them, effective regulatory mechanisms must be in place to ensure that these contribute towards agreed health sector strategies [18]. This paper examines these prerequisites for donor aid to be effective, but does not examine aid effectiveness as such.

## Methods

The analysis is both qualitative and quantitative. Over a 4-year period (2004–2007), retrospective evidence from four main sources was collected: 1) literature on aid management in Uganda, 2) key informant interviews guided by semi-structured questionnaires, 3) data trends on financial outlays for health activities, and 4) participant observations of key Sector Wide Approach (SWAp) processes. Information concerning aid management in Uganda was extracted using document review of published and non-published literature mostly generated as part of donor-government interactions, project evaluations, and special studies on aid [19–22] and the definition of terms used in the study is provided in Table 1 .

To supplement the documentary review, qualitative information was extracted from purposive in-depth interviews conducted with the aim of assessing the flow of aid for HIV programs in Uganda. In aggregate, 23 interviews were analyzed drawn from 8 institutions spanning central government and sector ministries, donor officials, and officials among recipient organizations providing health services. Additional qualitative evidence was drawn from participant observations made by the authors during key meetings and interactions between health sector officials and their donor partners. Most of these meetings were related to the annual assessments and monitoring of health sector performance as well as meetings for negotiating annual budget allocations by government and donor projects. Further observations are derived from the sector budget working group meetings that discuss resource mobilization and allocation and use issues for the sector. These are regular calendar events whose attendance are part of the job routine of the first author and frequently attended for research purposes by the second author. .

Trend data on the financial outlays by government and donor projects were extracted from administrative reports prepared for the purpose of Annual Health Sector Performance Reviews. These reports are prepared and agreed upon jointly by the government and health sector donors and represent the official form of donor-government accountability [23]. Additional trend data was extracted from the annual donor expenditure surveys that are the basis for monitoring donor project disbursements that are supporting health activities in Uganda. Likewise, HIV/AIDS survey reports that report donor project expenditure on HIV/AIDS and the Public Expenditure Reviews (PER) conducted by MoH were reviewed. These provide disaggregated expenditures on all public funds. Attempts were made to validate the data in the administrative reports by comparing with independent studies on aid in the Ugandan health sector where appropriate.

The assessment of pre-requisites for effectiveness of donor funds was restricted to donor funding through projects and GHI which contribute over 50% of public health sector expenditure. The limitation of this focus is acknowledged partly because greater alignment with the national budget support systems does create problems for analysts to tract its flow to the health sector or indeed specific inputs. Furthermore, this paper excludes funds raised for responding to emergencies/ epidemics such as Ebola or the pandemic H1N1 influenza.

## Results

### Levels of donor aid

**Figure 1:**
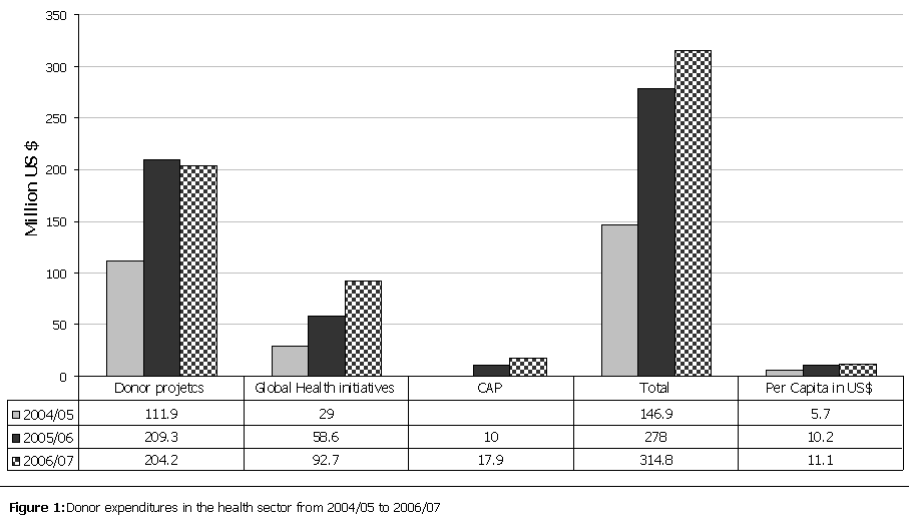
Donor expenditures in the health sector from 2004/05 to 2006/07

Significant increases in donor funds for the health sector have been noted over the years increasing from US$6 per capita in fiscal year (FY) 2004/05 to US$11 per capita in 2006/07 ( Figure 1 ). The highest increase over the previous year (over 80%) was registered between 2004/05 and 2005/06. This was largely from the GHI which had an increase of 58%.

### Predictability

Donors provide project budget figures at the beginning of the budget cycle and efforts are made as much as possible to discuss priorities to be funded between the donors and the MoH. Expenditures against budgets for the different development partners were compared ( Table 2 ). This analysis was restricted to those projects that are captured in the medium term expenditure framework (MTEF). We see large variance between the provided budgets and expenditure figures at the end of the FY ranging from 174% to 390%.

### Comprehensive budgeting and planning

**Figure 2:**
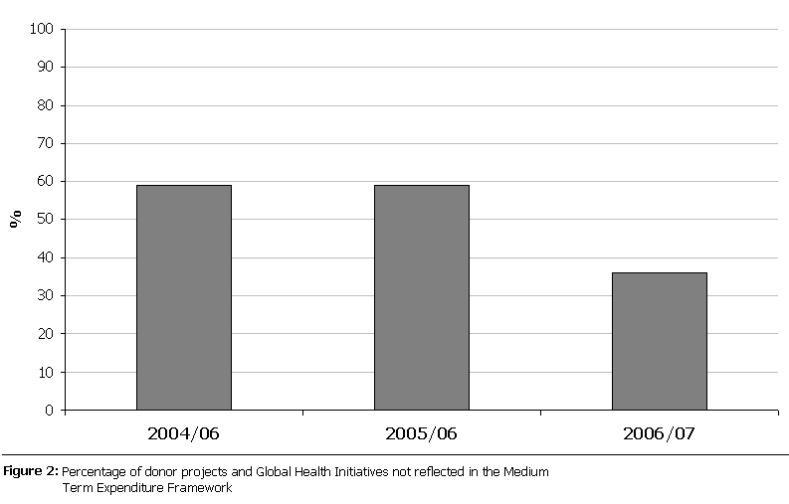
Percentage of donor projects and Global Health Initiatives not reflected in the Medium Term Expenditure Framework

Analysis was done to assess what percentage of donor project funding is off budget excluding emergency and private sector expenditures ( Figure 2 ). A significant proportion of donor project expenditures remain off budget, consistently over 50% except for FY 2006/07.

In order to put donor aid to effective use, it must be part of the routine budgeting and planning process to ensure that funds are allocated to agreed priorities. Concerns about lack of comprehensive financial information at the time of planning and budgeting were also expressed through qualitative findings as shown in the quote below:

*
“the donors have been at the planning table with this SWAP. They know what the needs are for the sector. They know the resource envelop. But the problem I see is that we plan for the small resource envelop (MTEF) but along the way Global Fund proposals come with huge funds or PEPFAR allocates its money after the plans are made. All these have different requirements - use different fiscal years and (have) different disbursement timetables. It becomes difficult to reconcile the national plan and the projects like Global Fund or PEPFAR.” (MOH Official)
*

### Channeling of donor funds

We note that funds are channeled through both public and private entities. Although private entities play a role in service delivery, they must be regulated and supervised to ensure that they are contributing to sector objectives. In FYs 2004/05 and 2006/07, 38% and 74% of donor project and GHI funding were channeled through the private sector respectively.

Looking at transfers through the private sector (these include facility-based Private not for Profit (PNFP), non facility-based PNFP and private for profit) we note that channeling of funds through facility-based PNFPs reduced significantly from 96% of transfers through the private sector in 2004/05 to 25% in 2006/07. Transfers are largely through the non facility-based PNFPs.

### Alignment to priorities

**Figure 3:**
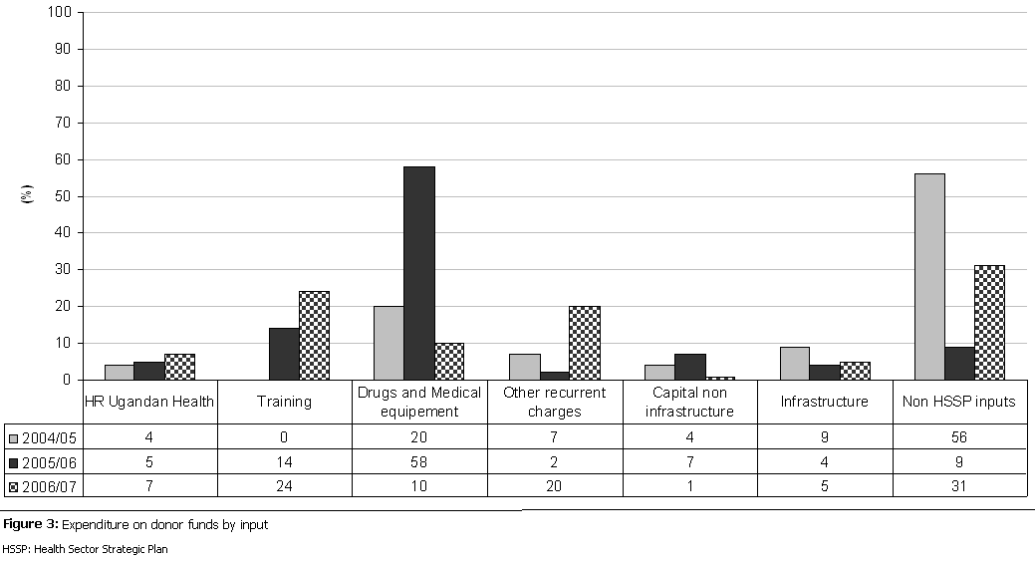
Expenditure of donor funds by inputs HSSP: Health Sector Strategic Plan

Donor funds must be properly aligned to sector priorities to ensure maximum benefits. Figure 3 shows the expenditure patterns of donor funds assessing the extent to which they are in line with key inputs costed in the Health Sector Strategic Plan (HSSP) II. HSSP II inputs include human resource for health for staff employed by the government and private health service providers, medicines and medical supplies, health infrastructure and equipment, training, recurrent costs core to health service provision like stationery, utilities, vehicle maintenance, feeding and bed linen in health facilities.

There is no specific pattern in expenditures by inputs; however, we note that expenditure on human resource remains meager given the fact it is the major constraint to service delivery. Expenditure on non HSSP II inputs, which include technical assistance and project management costs which are not direct inputs into delivery of HSSP II, reduced from 56% in 2004/05 to 31% in 2006/07. Although there is technical assistance (TA) critical to service delivery, a decision was made by the health sector not to include TA as a core input into HSSP II. Furthermore, if TA is to play the role it is envisaged, then there must a framework to identify TA needs, recruit the most appropriate person and ensure capacity building of local staff. An analysis done to review and analyze TA in the MoH showed that these were not in place [24].

We also see a bias in expenditures on medicines where three expensive commodities funded by donor funds have accounted for a larger percentage expenditure on medicines ( Table 3 ). Essential medicines include all medicines and medical supplies to address diseases affecting most of the population but exclude Artemisin-based Combined Therapy (ACTs) for malaria, antiretrovirals for HIV/AIDS, and pentavalent vaccine for immunization.

These expensive commodities were initially funded by the GHI, specifically GFATM for ACTs and ARVs and GAVI for the pentavalent vaccines. However, Uganda was affected by the reduced disbursement of funding from GHI due to mismanagement and the Ministry of Finance Planning and Economic development (MoFPED) had to identify funds from within the budget to sustain patients already on these life-saving medicines.

### Stewardship

The need for accountable governments and government leadership for good harmonization to happen was also emphasized as shown in the quote below.

*
“In reality, shortly after the Uganda Joint Assistance Strategy (UJAS) was adopted (2005), there was a shift in the environment in terms of several factors. For example, the perception of the reform agenda in Uganda changed which put the UJAS as an instrument for collective analysis and decision making under strain; and the performance of the social sectors began to slip which caused donors to hesitate about using government budget support to support the government, thus negatively effecting on the impact of the UJAS as a tool for alignment and harmonization. … What became clear in Uganda is that the role of Government in a joint programming exercise cannot be underestimated. The success of joint programming exercise largely depends on the genuine commitment of recipient countries to take full advantage of the potential benefits that UJAS can bring about. This can only be the case if recipient countries own these exercises and if they are not donor driven. In Uganda, donors have prepared the ground, but it is now time for government to take over.”* [25]

*
“USG has received a lot of bashing for not putting its (PEPFAR) money into the (Government) system (budget support). What most people do not understand is (that) US Government laws don’t allow us to give government money to do it’s job. US facilitate other players to help government where it needs help. Even if the (US) laws were not restrictive, the stories about misappropriation of Global Fund and GAVI monies would vindicate the model used by the US Government” (Donor Official)
*

### Fiscal sustainability

As developing countries continue to receive donor aid for health, they should also ensure mobilization of domestic resources. This does not seem to have happened ( Table 4 ) with no significant change in per capita expenditure on health from GoU as a source. Earlier financial years showed a similar trend with GoU per capita expenditure only increasing marginally from US $3.1 per capita in 2000/2001 to US$4.7 per capita in 2003/04 [10].

The GoU funding includes donor funding that is channeled through budget support. Donor funding to the GoU budget was 46% in 2004/05; 40% in 2005/06, and 41% in 2006/07 [26]. Reliance on donor funding is still an issue for the health sector given the fact that donor project expenditures contributed more than 50% of sector funding in 2004/05 (US$5.7 percapita compared to US$4.8 per capita from GoU) and close to 70% in 2005/06 (US$10.2 per capita compared to US$4.6 per capita from GoU) and 69% in 2006/07 (US$ 11 per capita compared to US $5.0 per capita from GoU).

## Discussion

### Level of donor aid

Donor aid has increasingly played as a significant role in financing health services in Uganda. Whether this will contribute to improvement of population health will need further follow up. For example, with increasing ODA, institutional deliveries have remained below 30% for the last four years [27], HIV prevalence has stagnated at 6% for the last four years [28], and outpatient attendance (OPD) has stagnated at 0.9 per capita since 2004/05 [10]. The task force on innovative international financing for health systems has emphasized that more money for health is as important as more health for the money [29].

### Predictability

We see a large variance between the provided budget and expenditure figures at the end of the FY. Several reasons account for the observed lack of predictability, including providing fewer figures at the budgeting time compared to what is spent and the MoH occasionally making *ad hoc* requests to donors to fund interventions within the FY. This huge variance negatively affects the comprehensive planning and budgeting process thus reducing the effectiveness of donor aid. Although this may appear favorable, it creates challenges having to absorb funds that were not anticipated at the planning stage and capacity to implement activities funded may not be ensured. Similar results of high volatility have been noted in other developing countries [30–31]. Lack of predictability has been enhanced by the probable nature of some funding modalities. The GFATM and GAVI are examples with probabilities at several stages from grant approval, periodic disbursements, and phase-two continuation of grants. These multiple probabilities create uncertainties in the planning and among implementers of the program [32–34].

### Comprehensive budgeting and planning

A significant proportion of donor project funds remain off budget. Monitoring of these funds has been noted to be problematic [4, 9]. Significant portions of off-budget donor aid, as documented in other developing countries [21], is associated with increased transaction costs such as midcourse re-planning.

### Channeling of funds

Increasing channeling of funds through NGOs has been noted in this analysis. Similarly increased spending through NGOs from 13% of DAH in 1990 to 25% in 2006 was also documented by Ravishankar et al [4]. Spending of donor aid through the private sector mainly requires capacity of MoH to regulate this sub sector. Although facility-based PNFP contribute slightly over 30% of health sector outputs [27], a larger proportion of funds is through private for-profit sector and non-facility-based PNFPs. Resource tracking studies have shown the poor expenditure patterns among non- facility-based PNFPs; the health expenditure tracking study showed that they spent over 70% of funding on administration [35]. Other studies have shown that most of the non facility-based PNFP lack the required capacity to implement health programmes and have weak management, accountability, and monitoring systems [36–37]. If channeling of funding through private for profit sector and non facility-based PNFPs is to effectively contribute to sector objectives, a coordination and regulatory framework should be put in place. In addition, the capacity of these subsectors needs to be built to strengthen their management systems, implement health programmes, and enable them to participate in key fora within the health sector.

### Alignment to priorities

Results have shown that alignment to sector priorities needs improvement as evidenced by a significant percentage expenditure on non HSSP II priorities. The gross under expenditure on human resource, which is a major challenge to implementation of health programmes, is a cause for concern. This partly stems from the weak processes to ensure alignment of donor funding to priorities. The PER for the health sector documented weak systems and processes for coordinated management and comprehensive monitoring of donor funded projects. Reporting was piece-meal and it was difficult to establish who the recipients of the funds were [9].Other studies have also shown low investments in general health system support not linked to specific diseases [4].

Preferential medicines expenditure on large expensive commodities has been shown in this analysis. Although these are life-saving commodities, they are largely funded by donor funds. The interruption of flow of these funds led to the government having to identify funding from within the budget which may translate into taking funding from other priorities. A large share of AIDS-related activities as a percentage of total donor support for health (exceeding 70%) in a number of developing countries has been documented in other studies [33]. Similarly, Ravishankar et al documented an increase in disbursements for HIV from 7% of official global DAH in 2000 to 23% in 2007 [4].

### Stewardship

We note that Uganda adopted the UJAS in 2005, but the shifts on the side of government negatively affected the already agreed framework. The weak leadership and institutions may be partly to blame for emergency of several donor specific administrative systems for information and logistics management. Given the slow bureaucracy in the public systems, donors may prefer to bypass the national systems and spend the money among the private sector for quick results. The implication is increased expenditure on non-HSSP objectives, poor alignment on sectoral expenditure priorities, and overall poor effectiveness of donor aid.

### Sustainability

Reliance on donor funding is still an issue for the health sector with insignificant increase in GoU per capita expenditure on health. Reduction in government spending on HIV/AIDS since the influx of donor spending was also noted in Kenya and Zambia [38]. Most of these donor projects are time-bound and given the stagnant GoU investment in health, achievements from these projects may not be maintained. Uganda running out of ARVs after a shortfall in aid has already been documented [39]. Concerns that the development assistance could drop have also been expressed [40–41]. Domestic resources need to be increased to ensure sustainability of donor-aided projects.

## Conclusion

We have showed that donor project and GHI funding for health is significant, but the perquisites for it to be effective need to be addressed. The faulty elements identified in this paper should be addressed in accordance with the Paris Declaration.

We recommend the following approaches (1) strengthen government institutions for coordinating and monitoring donor aid; (2) regulate administrative costs on donor aided projects to limit the wastage associated with these expenditures; (3) improve predictability through more consultative planning process to ensure that all priorities are agreed by majority of stakeholder to minimise ad-hoc requests during the course of the FY. Provision of reliable figures on likely funding and ensuring timely disbursement in line with the budget should be ensured; (4) build the capacity of the private sector in service delivery, management and participation in key processes within the sector. Furthermore, there is need to put in place a regulatory and monitoring framework to ensure that these contribute to sector objectives; (5) establish an agreed approach to measuring additionality to donor aid from domestic resources.

## Figures and Tables

**Table 1:** Definition of terms

** Predictability **	The extent to which donor aid projected to be acquired in the future is actually realized
** Comprehensiveness **	The extent to which donor aid finances the essential health package as a whole
** On budget **	The proportion of donor aid in the government budget
** Off budget **	The proportion of donor aid outside the government budget
** Fungibility **	The extent to which donor aid can be reallocated according to changing local needs
** Absorptive capacity **	The ability of the country to use available donor aid at its disposal
** Stewardship **	Leading and steering of health policy and policy direction in the agreed interests of the country
** Donor aid coordination **	Harmonizing donor aid to address priority needs of the country as agreed in a national strategic plan or policy
** Sustainability **	The extent to which the health and activities previously funded by donor aid can be maintained over medium and long periods with minimal external support

**Table 2:** variances in donor project funding figures; budgets Vs expenditures; Million US$

** Financial year **	** Donor project Budget figures in MTEF **	** Donor expenditure survey **	** Difference between expenditure and budget **	** Performance against MTEF budget **
2004/05	84.59	146.91	62.32	174%
2005/06	147.06	277.95	130.89	189%
2006/07	80.70	314.80	234.10	390%

In 2003/04, there marked under spending on the Global Fund against AIDS, Tuberculosis and Malaria, following suspension of the Project Management Unit. Source; GoU, MoH, Annual Health Sector Performance Report 2005, 2006, 2007. MTEF: Medium Term Expenditure Framework

**Table 3:** Disease specific focus of donor funding; spending against expensive commodities in US$ per capita (% of total medicines expenditure)

	** 2004/05 **	** 2005/06 **	** 2006/07 **
Essential medicines	1.87	1.00	1.67
Vertical and specialized (ACT’s, ARV’s, Pentavalent vaccine, ITNs) funded by donor aid	1.24 (40%)	3.00 (75%)	2.3 (56%)
** Total **	** 3.11 **	** 4 **	** 4.06 **

ACT: Artemisin-based Combined Therapy, ARV’s: Antiretrovirals, ITNs: Insecticide treated nets

**Table 4:** Has the Government of Uganda increased its allocation to health

** Year **	** Per capita expenditure in US$ **	** As % of expenditure **	** Increase on the previous year **
** 2004/05 **	4.8	10%	5%
** 2005/06 **	4.6	9%	6%
** 2006/07 **	5.0	10%	NA

Source: computed from expenditure figures for Government of Uganda funding
